# Stereotactic radiosurgery for spinal metastases: a literature review

**DOI:** 10.1590/S1679-45082013000200020

**Published:** 2013

**Authors:** Andrei Fernandes Joaquim, Enrico Ghizoni, Helder Tedeschi, Eduardo Baldon Pereira, Leonardo Abdala Giacomini

**Affiliations:** 1 Universidade Estadual de Campinas, Campinas, SP, Brazil.

**Keywords:** Radiosurgery, Neoplasm metastasis/therapy, Spinal neoplasms/radiotherapy

## Abstract

**Objective:**

The spine is the most common location for bone metastases. Since cure is not possible, local control and relief of symptoms is the basis for treatment, which is grounded on the use of conventional radiotherapy. Recently, spinal radiosurgery has been proposed for the local control of spinal metastases, whether as primary or salvage treatment. Consequently, we carried out a literature review in order to analyze the indications, efficacy, and safety of radiosurgery in the treatment of spinal metastases.

**Methods:**

We have reviewed the literature using the PubMed gateway with data from the MEDLINE library on studies related to the use of radiosurgery in treatment of bone metastases in spine. The studies were reviewed by all the authors and classified as to level of evidence, using the criterion defined by Wright.

**Results:**

The indications found for radiosurgery were primary control of epidural metastases (evidence level II), myeloma (level III), and metastases known to be poor responders to conventional radiotherapy – melanoma and renal cell carcinoma (level III). Spinal radiosurgery was also proposed for salvage treatment after conventional radiotherapy (level II). There is also some evidence as to the safety and efficacy of radiosurgery in cases of extramedullar and intramedullar intradural metastatic tumors (level III) and after spinal decompression and stabilization surgery.

**Conclusion:**

Radiosurgery can be used in primary or salvage treatment of spinal metastases, improving local disease control and patient symptoms. It should also be considered as initial treatment for radioresistant tumors, such as melanoma and renal cell carcinoma.

## RESUMO

**Objetivo:**

A coluna vertebral é o local mais comum de metástases ósseas. Uma vez que a cura não pode ser obtida, o controle local e o alívio dos sintomas é a base do tratamento, sendo este fundamentado no uso de radioterapia convencional. Recentemente, a radiocirurgia espinhal foi proposta para o controle local das metástases na coluna, seja como tratamento primário ou de resgate. Dessa forma, realizamos uma revisão da literatura para analisar as indicações, a eficácia e a segurança da radiocirurgia no tratamento das metástases da coluna.

**Métodos:**

A revisão de literatura foi realizada no portal PubMed – dados da biblioteca MEDLINE, sobre os estudos relacionados ao uso da radiocirurgia no tratamento para metástases ósseas na coluna vertebral. Os estudos foram revisados por todos os autores e classificados quanto ao nível de evidência, utilizando critério definido por Wright.

**Resultados:**

As indicações encontradas para radiocirurgia foram: controle primário de metástases epidurais (nível II de evidência), mieloma (nível III) e metástases sabidamente pouco responsivas à radioterapia convencional – melanoma e carcinoma de células renais – (nível III). A radiocirurgia espinhal também foi proposta para o tratamento de resgate após falha da radioterapia convencional (nível II). Existe ainda alguma evidência quanto à segurança e a eficácia da radiocirurgia em casos de tumores metastáticos intradurais extramedulares e intramedulares (nível III), e após cirurgias de descompressão e estabilização da coluna.

**Conclusão:**

A radiocirurgia, portanto, pode ser usada no tratamento primário ou de resgate de metástases espinhais, melhorando o controle local da doença e dos sintomas dos pacientes. Deve ainda ser considerada como tratamento inicial para tumores radiorresistentes, como melanoma e carcinoma de células renais.

## INTRODUCTION

The spine is the most common site for bone metastases^(1)^. Spinal involvement occurs in up to 40% of patients with cancer during progression of the disease, with 5 to 10% of these patients developing epidural compression at some point of their progression^(2)^. More than 90% of the spinal metastases are located extradurally, whereas about 5% are intradural and less than 1% are intramedullar^(3)^. Symptomatic compression occurs more frequently in the thoracic spine (50 to 70%), followed by the lumbar spine (20 to 30%) and the cervical spine (10 to 30%)^(4)^. This probably occurs because the thoracic canal has the smallest diameter and the largest number of vertebrae in the spine. The posterior half of the vertebral body is the most commonly involved^(4)^, spreading latterly to other regions (anterior body, lamina, and pedicles). Also important is the fact that by the time of diagnosis, multiple lesions can be found at noncontiguous levels in up to 40% of the patients. Regarding histology, up to 50% of the metastases come from one of the three following cancers: breast, lung, or prostate^(1)^.

Since spinal metastases suggest a non-controlled tumor, treatment is mainly based on palliation and local disease control. Although in selected cases chemotherapy can be used, radiotherapy, with or without concomitant surgery, remains the main treatment modality^(5,6)^. Surgical techniques have progressed substantially in the last years, allowing circumferential decompression of the spinal cord and spinal fixation for early stabilization^(6)^. The main goals of surgery are spinal stabilization, restoration, and maintenance of the global alignment and decompression of neural structures, especially in radioresistant tumors^(5)^. Surgery can also be an option in unknown primary tumors (when a tissue sample is necessary) or in cases where local pain persists after other treatment modalities have been used. However, the surgical option should only be considered for patients with good performance scores, since it may entail serious morbidity and possible medical complications. Prognostic scoring systems, like the one proposed by Tokuhashi et al. and Tomita et al., are useful in surgical decision-making^(7,8)^.

### Role of radiation therapy in spinal metastases

Since surgery is only considered in selected cases, conventional external beam radiotherapy (CER) remains the mainstay treatment modality for spinal metastases. The total dose of radiation ranges from 25 to 40Gy, fractionated daily into 8 to 20 doses^(6)^. A broad margin is used in the radiation field, typically one or two vertebral segments above and two segments below the affected level in order to compensate for internal vertebral motion. This technique exposes healthy tissue to radiation, including the sensitive spinal cord. The dose of radiation is fractioned to allow recovery of the normal tissue, thus improving tolerance^(6)^.

Spinal stereotactic radiosurgery (SRS) is a more recently developed type of radiation therapy that delivers a high dose of radiation to the tumor, whilst minimizing the amount delivered to the healthy neighboring tissues. The target is defined by high-resolution stereotactic imaging, and requires rigid spine immobilization to offer a precise conformal dose, using frame or frameless techniques, based on external immobilization devices with fiducial markers. Treatment is fractionated into fewer sessions (one to five), with total doses ranging from 8 to 30Gy. When one session is used, the term “radiosurgery” must be preferentially used, whereas “fractionated stereotactic radiosurgery” is better used when multiple sessions are performed. Early versions of SRS in the spine required surgical fixation of frames in the spinous processes to avoid movement artifacts. The novel systems are frameless, based on internal skeletal anatomy, and implanted fiducial points, or infrared surface markers, with near real-time images for motion correction. Radiation intensity modulation increases the conformity of radiation to the tumor, minimizing the dose to normal tissue^(6)^. However, the effectiveness, safety, precise indications, and long-term evaluation regarding disease control of the effects of SRS remain unclear.

## OBJECTIVE

To perform a literature review of the indications, safety, and efficacy of spinal radiosurgery when compared to conventional external radiotherapy for the treatment of spinal metastases.

## METHODS

### Search strategy

A literature review using the PubMed gateway of the MEDLINE database was performed, with no time restriction, until September, 2012. The following key-words were queried, singly or in combination: “radiosurgery”, “stereotactic”, “spinal metastases”, “spinal cancer”, “treatment”, “indications”, “radiation therapy”, and “extracranial”.

### Study selection

We included clinical papers of patients who received radiosurgery for the treatment of spinal metastases.

We excluded non-English language papers, literature reviews, case reports, and papers not focused on radiosurgical treatment of spinal metastases.

### Data extraction

One hundred and twenty-four studies were reviewed and scrutinized according to study design (retrospective case series, prospective studies, and randomized control trials), patient characteristics, and primary findings by four independent researchers (AFJ, EG, HT, LG). After analysis of the four authors, reselection was made and a total of 31 studies were selected and included in our review.

### Measurements and outcome evaluation

The results of the studies were grouped according to their main purpose: pain control, radiological control, overall survival, comparison of treatment modalities (SRS *versus* CER *versus* surgery), indications for spinal radiosurgery, and methods of evaluating disease control and response to treatment.

### Evaluation of the level of evidence

The studies were classified according to the level of evidence for therapeutic studies, defined by Wright et al.^(9)^.

1. Higher quality randomized controlled trial.
2. Lesser quality randomized controlled trial; prospective comparative study.
3. Case-control study; retrospective comparative study.
4. Case series.
5. Expert opinion.

## RESULTS

Out of 31 selected studies, a total of 2,241 patients were treated for spinal metastases. All papers are summarized on chart 1.

**Chart 1:** Selected studies after a systematic review using the MEDLINE database

	Study type	Radiosurgery status/indication	n	Dose	Results
Ahmed et al.^(10)^	Prospective case series	Primary and reirradiation therapy for spinal metastases	66 patients (85 lesions)	24Gy (10-40Gy) (median 3 fractions, range to 1-5)	The mean actuarial survival at 12 months was 52.2%
					7 patients had both local and marginal failure, 1 patient experienced marginal but not local failure, and 1 patient had local failure only Actuarial local control at 1 year was 83.3% and 91.2% in patients with and without prior RT, respectively
					No Grade 4 toxicities were reported
Amdur et al.^(11)^	Prospective case series	Primary and reirradiation therapy for spinal metastases	21 patients (9 patients; no prior radiotherapy and 12 patients with prior radiotherapy)	15Gy (no prior spinal radiotherapy) 5Gy (prior spinal radiotherapy)	No late toxicities
					3 patients experienced radiographic evidence of vertebral body compression in field
					43% experienced pain relief
					1-year progression-free survival was 5% with 60% of patients dead by 1 year
					In patients with and without prior radiotherapy, they achieved the target-coverage goal in 91% and 95%, respectively
Benzil et al.^(12)^	Case series	Primary and reirradiation therapy for spinal metastases and primary tumors	31 patients (35 lesions); 26 metastases and 4 intradural and 5 extradural tumors	Mean single dose 2.68Gy and mean total dose 6.89Gy for intradural tumors	Significant pain relief was achieved in 32/34 treated tumors
					Pain relief was achieved with a single dose as low as 5Gy
					2 patients experienced transient radiculitis (both with a BED >60Gy) No patient experienced other organ toxicity
Chang et al.^(13)^	Case series	Primary radiosurgery for spinal metastases	63 patients	27-30Gy (three 9Gy fractions and five 6Gy fractions)	No neurological complications (median of 21.3 months of follow-up)
					1-year tumor progression-free was 84%
					Mild symptoms of toxicity (nausea, vomiting, and diarrhea)
Chang et al.^(14)^	Case series	Primary and reirradiation therapy for spinal metastases	129 patients (53 reirradiation)	16-39Gy in 1-5 fractions	Pain relief in 91%
					In 108 lesions, 75 decreased or stable mass size
Chang et al.^(15)^	Case series	Primary and reirradiation therapy for spinal metastases	54 retreatment and 131 initial SBRT	Mean radiation doses to tumor margin 51.1Gy 2/10 (retreatment) and 50.7Gy 2/10 (initial treatment)	Mean progression-free period was 23.9 months (overall); 18 months (retreatment) and 26 months (initial treatment)
					Radiological control rates were about 95% at 6 months and up to 80% at 12 months
					No radiation myelopathy
De Salles et al.^(16)^	Case series	Radiosurgery for spinal metastases and benign spinal tumors	14 patients (22 lesions); 11 patients with metastases, 2 neurofibromas, and 1 meningioma	A mean dose of 12±2.7Gy (range 8-21Gy) x 13 received singledose stereotactic radiosurgery	Mean follow-up period was 6.1±3.9 months (range 1-16 months) 3 patients became pain-free and 4 experienced considerable relief Weakness improved in 2 patients with this preoperative symptom and the asymptomatic patients remained asymptomatic
					4 lesions decreased in size, 5 remained stable, 7 progressed, and 6 were not followed (2 patients died before follow-up)
					4 patients in all died, 3 of systemic disease and 1 of thoracic lesion progression
					No complications were observed
Garg et al.^(17)^	Prospective case series	Primary radiosurgery for spinal metastases	61 patients (63 tumors)	16-24Gy (single fraction)	Mean follow-up of 20 months
					Actuarial 18-month imaging local control rate for all patients was 88%
					Actuarial 18-month overall survival rate for all patients was 64% with a median survival for all patients of 30 months
					No significant differences in outcomes were noted with respect to tumor histology or dose
					The data support an expanded indication for spinal radiosurgery as first-line treatment
Levine et al.^(18)^	Case series	Primary radiosurgery for primary spinal sarcomas and metastases	24 patients	30Gy	7 patients definitively treated; 2 complete regression, 3 partial regression, and 2 late recurrence re-treated
					7 had surgery + SRS; 5 cases of total tumor control with mean follow-up of 43.5 months
					10 patients with metastases; all died; mean 11.1 months of survival; 80% pain relief
					No myelitis
Martin et al.^(19)^	Case series	Primary (14 lesions) or metastatic (39) spinal lesions	41 patients (53 lesions)	8-30Gy (1-3 fractions)	Median follow-up of 11.1 months
					59% of patients experienced no acute side effects from treatment; there were 3 cases of acute grade 3 toxicity
					Local control and overall survival were 91 and 65%, respectively
					Pain improvement was seen in 75% of symptomatic metastases at 6 months post-treatment
Patel et al.^(20)^	Case series	Primary irradiation of spinal metastases	117 patients (154 lesions)	Single fraction comparing whole (W) *versus* partial (P) contour approach of the vertebral body	W Group had a lower re-treatment rate (11% for W Group *versus* 18.6% for P Group; p=0.285)
					Prior surgery status (β=1.953; OR=7.052; p <0.001) was correlated to the re-treatment rate
					The 2-year survival was 25.7% in W Group and 20.9% in P Group (p=0.741)
					They concluded that contouring the whole vertebral body for stereotactic body radiation therapy treatment of metastatic spinal lesions shows potential benefits by reducing the risk of recurrence, improving symptomatic relief, and providing improved local tumor control
Ryu et al.^(21)^	Case series	Primary radiosurgery for spinal metastases	49 patients (61 lesions)	10-16Gy (single dose)	Complete/partial relief in 85%
					Relapse of pain in 7%
					5% of radiologically adjacent spine metastases
Wang et al.^(22)^	Case series	Primary irradiation of spinal metastases	149 patients (166 lesions)	27-30Gy (3 doses)	Median follow-up of 15.9 months
					Pain control improved from 26-54% at 6 months
					Progression-free survival after SBRT was 80.5% at 1 year
Gerszten et al.^(23)^	Case series	Primary and reirradiation therapy for renal cell carcinoma metastases	48 patients with 60 RCC metastases (42 lesions were reirradiated)	Tumor dose range to 17.5-25Gy (mean 20Gy); single dose	Follow-up for 14-48 months (median 37 months)
					No radiation-induced toxicity occurred during the follow-up period
					Axial and radicular pain improved in 34 (89%) of 38 patients who were treated primarily for pain
					Tumor control was demonstrated in 7 of 8 patients treated primarily for radiographically documented tumor progression
					6 patients required open surgical intervention for tumor progression that had caused neurological dysfunction after radiosurgery
Gerszten et al.^(24)^	Case series	Primary and reirradiation therapy for melanoma spinal metastases	28 patients (36 lesions in 23 patients with previous external beam irradiation)	Maximum dose was 17.5-25Gy (mean 21.7Gy); single fraction	Follow-up period of 3-43 months (median 13 months)
					No radiation-induced toxicity occurred during the follow-up period
					Axial and radicular pain improved in 27 of 28 patients (96%) who were treated primarily for pain
					Long-term tumor control was seen in 3 of 4 cases treated primarily for + tumor progression. Two patients went on to require open surgical intervention for tumor progression resulting in neurological deficit
Choi et al.^(25)^	Case series	Reirradiation therapy for spinal metastases (median previous spinal cord dose of 40Gy)	42 patients (51 lesions)	Median dose of 20Gy (range 10-30Gy) in 1-5 fractions (median 2)	Median follow-up of 7 months (range: 2-47 months)
					The Kaplan-Meier local control and overall survival rates at 6/12 months were 87%/73% and 81%/68%, respectively
					Time to re-treatment of ≤12 months and the combination of time to re-treatment of ≤12 months with an SSED of <15Gy (10) were significant predictors of local failure on univariate and multivariate analyses
					1 patient (2%) experienced Grade 4 neurotoxicity
Gagnon et al.^(26)^	Case series (case control study)	Reirradiation therapy for breast cancer spinal metastases	18 patients treated - recurrent metastases (reirradiated) compared with 18 who received conventional RDP	Doses ranging from 21-28Gy (3-5 fractions)	Both groups were comparable along all matching dimensions and in performance status before treatment
					Outcomes of treatment were similar for patients in both groups; ambulation, performance status, and pain worsened similarly across groups post-treatment
					Survival and the number of complications appeared to favor the CyberKnife group, but the differences did not reach statistical significance
					Salvage CyberKnife is efficacious
Klish et al.^(27)^	Prospective case series	Reirradiation for previously irradiated spinal metastases	58 patients	Adjacent level disease after irradiation of the involved vertebral body	Avoid the historic irradiation of 1-2 vertebral bodies above/below the involvement using EBRT
					Multiple level of SRS irradiation is unnecessary
					3% of failure compared with <5% of isolated failures of the unirradiated adjacent vertebral body
Koyfman et al.^(28)^	Case series	Reirradiation for previously irradiated spinal metastases	149 patients (208 lesions)	14Gy(median dose ranging from 10-16Gy)	Median follow-up was 8.6 months, and median survival was 12.8 months Recurrence occurred in 26 (12.5%) treated lesions, at a median time of 7.7 months after conventional radiotherapy
					Patients with paraspinal disease at the time of conventional radiotherapy (20.8% *versus* 7.6% of patients; p=0.02), and those treated with <16Gy (16.3% *versus* 6.3% of patients; p = 0.14) had higher rates of recurrence
Mahadevan et al.^(29)^	Case series	Reirradiation for previously irradiated spinal metastases	60 patients	8Gy ×3=24Gy (far from the cord) 5 to 6Gyx5=25 to 30Gy (near the cord)	9 months median progression-free survival
					93% had stability
					65% had pain relief
					7% disease progression
					No toxicity
Nikolajek et al.^(30)^	Case series	Reirradiation for previously irradiated spinal metastases	54 patients (70 lesions)	Median radiosurgery dose: 1 × 18Gy(range 10-28Gy) to the median 70% isodose single fraction	Median follow-up of 14.5 months
					The actuarial rates of freedom from local failure at 6/12/18 months were 93%, 88%, and 85%, respectively
					In 6 out of 7 patients worse sensory or motor deficit after SRS was caused by local or distant failures (diagnosed by CT/MRI)
					1 patient with metastatic renal cell carcinoma developed a progressive complete paraparesis 1 year after the last treatment at lumbar level L3
Sahgal et al.^(31)^	Case series	Primary and reirradiation for previously irradiated spinal metastases	39 patients (60 lesions) 23 lesions were unirradiated 37 were reirradiated (31 had image tumor progression)	Median total dose prescribed was 24Gy in 3 fractions	Median survival time measured was 21 months (95%CI=8-27 months). 2-year survival probability was 45%
					Median tumor follow-up for the unirradiated and reirradiated group was 9 months (range: 1-26) and 7 months (range: 1-48) respectively
					8 of 60 tumors have progressed, and the 1- and 2-year PFP was 85% and 69%, respectively
					For the salvage group the 1-year PFP was 96%
					In 6 of 8 failures the minimum distance from the tumor to the thecal sac was <or=1mm
					39/60 had >or=6 months follow-up and no radiation-induced myelopathy or radiculopathy occurred
Sahgal et al.^(32)^	Case series	Reirradiation for previously irradiated with external beam radiation	19 patients (5 with radiation myelopathy - RMI and 14 without it - no-RMI)	Mean of 20Gy in the no-RMI group *versus* 1-5 fractions 67.4 in the RMI group	SBRT given at least 5 months after conventional palliative radiotherapy with a reirradiation dose of 20-25Gy (2/2) appeared to be safe, provided the total dose does not exceed approximately 70Gy (2/2)
Sheehan et al.^(33)^	Case series	Spinal radiosurgery using a helical Tomotherapy	40 patients (110 tumors); range 1-6 tumors per patient 23 (57.5% underwent previous surgery	Mean radiosurgical dose was 17.3Gy (range: 10-24Gy)	Mean follow-up duration of 12.7 months (range: 4-32 months)
					Decreased or stable tumor volume was seen in 90 (82%) of the tumors treated
					Pain improvement in 34 patients (85%)
Shin et al.^(34)^	Case series	Intradural extramedullar and intramedullar spinal metastases	9 patients	13.8Gy (10-16Gy)	80% improved symptoms
					10% worsened
					No radiation toxicity
Jin et al.^(35)^	Retrospective case series	Radiosurgery for epidural myeloma	24 patients	10-18Gy (16Gy)	81% had complete radiographic response
					86% pain control
					71% of improvement in neurological symptoms
Massicote et al.^(36)^	Case series	Primary irradiation after minimally invasive spinal surgery for unstable spinal metastases	10 patients		8 patients were symptomatic at baseline
					The median follow-up was 13 months (range: 3-18)
					Following surgery, the median time to SBRT treatment planning was 6.5 days and subsequent median time to treatment was 7 days
					Local control was observed in 7 of the 10 patients. Improvements in VAS, ODI, and QOL were observed post-SBRT
Moulding et al.^(37)^	Case series	Surgical decompression and stabilization for epidural compression followed by spinal radiosurgery	21 patients (20 tumors; 95% were considered highly radioresistant to conventional external beam radiotherapy)	18-24Gy (median 24) single dose Planned target volume received a high dose (24Gy) in 16 patients (76.2%), and a low dose (18 or 21Gy) in 5 patients (23.8%)	15 (72%) of 21 patients died, and in all cases death was due to systemic progression as opposed to local failure
					The median overall survival after radiosurgery was 310 days One patient (4.8%) underwent repeat surgery for local failure and 2 patients (9.5%) underwent spine surgery for other reasons
					Local control was maintained after radiosurgery in 17 (81%) of 21 patients until death or most recent follow-up, with an estimated 1-year local failure risk of 9.5%
					Of the failures, 3 of 4 were noted in patients receiving lowdose radiosurgery
					Patients receiving adjuvant stereotactic radiosurgery with a high dose had a 93.8% overall local control rate (15 of 16 patients), with a 1-year estimated failure risk of 6.3%
Garg et al.^(38)^	Prospective case series	Reirradiation therapy for spinal metastases	59 patients (63 tumors)	30Gy (5 fractions; 6Gy) 27Gy (3 fractions; 9Gy)	1-year overall survival in 76% with local control
					92% freedom from neurological injury
					81% of the tumors within 5mm of the spinal cord developed cord compression
Gerszten et al.^(39)^	Prospective nonrandomized cohort study	Primary and reirradiation therapy for spinal metastases	500 cases	Maximum intratumoral dose range from 12.5-25Gy (mean 20Gy) (single fraction)	Long-term pain improvement occurred in 290 of 336 cases (86%)
					Long-term tumor control was demonstrated in 90% of lesions treated with radiosurgery as a primary treatment modality and in 88% of lesions treated for radiographic tumor progression 27 of 32 cases (84%) with a progressive neurologic deficit before treatment experienced at least some clinical improvement
Haley et al.^(40)^	Prospective case series	Compare the efficacy and cost effectiveness of EBRT versus SRS	44 (22 received EBRT and 22 SRS)		EBRT cost 29-71% of the SRS treatment, had more acute toxicity (but self-limited and with low grade), and more of them need further intervention (surgery/kyphoplasty)
					No late complication in either groups
					Similar pain relief

### Indications

Primary radiosurgery for treatment of epidural spinal metastases was reported in 13 studies, including spinal sarcomas. These studies were classified as level II and level III of evidence^(10–22)^. Two studies reported the efficacy and safety of radiosurgery in tumors with known low response to CER, and radioresistant tumors such as melanoma and renal cell carcinoma^(23,24)^.

SRS as salvage treatment, after failure of previously irradiated epidural spinal metastases with CER, was reported in 15 studies^(10–12,14,15,19,25–33)^ (levels II and III).

The use of spinal radiosurgery for treatment of intradural extramedullary and intramedullary spinal metastases and for epidural compression of myeloma was reported in one study each (level III)^(34,35)^.

Radiosurgery following surgery for decompression and stabilization was reported in three studies (level III)^(33,36–37)^.

### Dose intensity and number of fractions

The dose obtained in the studies for epidural metastases ranged from 8 to 30Gy (different low doses were used for intradural tumors)^(12)^, in one to five fractions. Moulding et al. considered that when lower doses were used (18 to 21Gy), worse local control was obtained compared to higher doses (18-24Gy). Some authors administered the same doses for all patients^(18)^, whereas others reported that factors such as prior conventional radiotherapy, distance of the epidural metastases from the cord^(29)^, and tumor histology^(23)^, among others, were used to individualize the dose. It also should be mentioned that lower doses were used for intradural and intramedullar metastases as well as for salvage treatment after prior conventional radiotherapy, in order to avoid late radiation myelopathy.

### Pain control

Although the lack of standardized outcome measurements in the reviewed studies precluded deep or statistical analyses, pain was substantially controlled (up to 90%) in many series, suggesting good efficacy in decreasing pain symptoms^(10–12,14,16,19,23,24,29,33,35,38,39)^. A potential advantage of SRS is the time required to obtain some pain relief, as early as 24 hours, probably quicker than CER^(21)^. However, it is important to emphasize that the methods utilized for pain evaluation in different papers were very heterogeneous, which precluded a uniform analysis (verbal analysis scale, and subjective measurements of pain relief, among others). In some tumors considered radioresistant to CER, pain control was achieved in 96% of the patients with melanomas and in 89% of the patients with renal cell carcinoma^(23,24)^. However, we also observed that low rates of pain control (43%) were obtained in a series using a lower dose of SRS^(11)^.

### Toxicity

Radiation-related toxicity was mild in almost all series, with rare reports of severe toxicity^(10,12–13,29,31)^. Transient radiculitis was reported in 2 of 31 patients of the Benzil et al. series^(12)^. Three patients of 41 in the cases published by Martin et al. presented with toxicity grade 3(19). These rarely reported cases of moderate toxicity in all the cases reviewed suggested that SRS is a safe procedure.

### Local disease control

The efficacy of spinal radiosurgery in local disease control was assessed clinically and radiologically in some studies. Chang et al. reported radiological control of spinal metastases in 90% of the patients at 6 months and in 80% at 12 months, similar to Garg et al., who obtained an imaging control rate of 88% of the patients after 18 months of follow-up^(15,17)^. Sheehan et al.^(33)^ also reported decreased or stable tumor volume in 82% of the patients treated (mean of 12.7 months of follow-up). Some papers also reported improvement in neurological deficits after treatment with SRS^(16,25,39)^.

Regarding tumor recurrence, some interesting information was obtained: Koyfman et al. reported that the presence of paraspinal disease at the time of CER (prior to SRS) was associated with high rates of local recurrence (p=0.02)^(28)^. A dose lower than 16Gy was also associated with local recurrence (p=0.14). Regarding the radiation target, Klish et al. evaluated a prospective series of 58 patients with good disease control and no damage to healthy tissues, and concluded that irradiation one or two levels above or below the involved segment is not necessary when SRS is performed^(27)^.

### Cost-effectiveness of CER *versus* SRS

In one prospective case series, Haley et al.^(40)^ compared the efficacy/cost- effectiveness of CER *versus* SRS. At 1-month follow-up, they reported that both methods have similar outcomes regarding pain relief, but CER costs about 29 to 71% less than the SRS treatment. However, CER has a high rate of acute toxicity (although it was self-limited and with low intensity) and required more interventional procedures (surgery, kyphoplasty etc.) at the level of the treated spinal. Neither method had late complications.

## DISCUSSION

The ideal treatment of spinal metastases would be one that obtains cure without adding excessive morbidity. However, since no treatment has been proven to increase life expectancy, the main objectives of treatment remain local disease control, pain relief, reestablishment of a normal neurological status, and avoidance of spinal deformity/instability. Surgery is reserved for an unstable spine, to obtain tissue samples, for pain relief in cases of instability, or for urgent decompression. Regardless of the indication for initial surgical treatment, almost all patients should be referred to radiation therapy treatment.

As shown in our data, SRS was efficient in controlling pain in many series^(10–12,14,16,19,23,24,29,33,34,38,39)^. Most of the studies showed pain control of up to 90%, even for radioresistant tumors such as melanomas and renal cell carcinoma. Since a cure is not possible, the palliative effect of SRS in pain control has been one of the main reasons for radiation treatment in these patients.

Clinical stability and radiological disease control were obtained in up to 95% of the series^(15,17,33)^. The efficacy of local control decreased with time, but most of the patients died from progression of the systemic disease before local recurrence^(37)^. The presence of paraspinal disease increases local recurrence probably because it is associated with late stages of cancer and a more systemic disease. This radiological control obtained with SRS can decrease or stabilize tumor volume, improving the neurological status in some series^(16,35,39)^. All these features confirm SRS as an efficient method for treating spinal metastases regarding local control.

Ideally, an efficient method that improves the patient's symptoms should not carry high morbidity rates. In this context, the studies assessed in our review with more than 2,000 patients did not demonstrate severe toxicity related to SRS. Moreover, when reported, toxicity was mild and self-limited, attesting to the safety of SRS in treatment of spinal cancer^(10,12,13,29,32)^.

Since safety and efficacy are warranted, the oncology group needs to individualize the patient's treatment and decide between SRS and CER. Although both methods require certainty of the diagnosis, SRS has its best indications for patients with more restricted spinal/ paraspinal metastases (involving one to two spinal segments); on the other hand, CER is more efficient for multiple level spinal involvement^(34)^. As stated before, although costs favor the indication of CER^(40)^, this assertion does not really reflect the truth, especially for patients who live far from radiotherapy centers, as SRS requires less fractions (1 to 5) compared to CER (8 to 20 daily fractions).

Although many questions are not yet solved, SRS has proven to be an efficient and safe alternative to CER for treating patients with restricted spinal metastases. The best indication for using SRS as a primary treatment of spinal metastases is likely for disease restricted to one or two levels of a known poor responder to CER, such as renal cell carcinoma and melanomas. Another potentially good clinical scenario for SRS is in cases in which CER has failed to achieve local disease control.

## CONCLUSION

SRS can be used for the treatment of epidural spinal metastases (level II of evidence) and spinal myeloma (level III), especially in tumors known to be poor responders to CER (such as melanoma or renal cell carcinoma) (level III). SRS is also indicated for salvage treatment when CER fails to control disease (level II), as reirradiation of patients without any other potential treatment available. There is some evidence (level III) of the efficacy and safety of SRS in the treatment of intradural extramedullary and intramedullary spinal metastases. The same is true for its use after surgical procedures for canal decompression and stabilization (level III). Further prospective studies are necessary to compare the potential benefits of SRS over CER.
